# Supplementary material for: Empirical methods for controlling false positives and estimating confidence in ChIP-Seq peaks
Source: BMC Bioinformatics. 2008 Dec 5;9:523. doi: 10.1186/1471-2105-9-523 (PMC2628906; doi:10.1186/1471-2105-9-523)
Supplement: Additional File 1 — A variety of html documents from the USeq web site detailing the available applications, their best usage, output file type descriptions, command line menus, etc. [file 1471-2105-9-523-S1.zip › USeqUserGuides/JavaDoc.zip!/JavaDoc/trans/tpmap/MummerMapper.html]

MummerMapper


|  |  |  |
| --- | --- | --- |
| **Overview** \| **Package** \| **Class** \| **Use** \| **Tree** \| **Deprecated** \| **Index** \| **Help** | |  |
| **PREV CLASS**   **NEXT CLASS** | **FRAMES**    **NO FRAMES**     **All Classes** |  |
| SUMMARY: NESTED \| FIELD \| CONSTR \| METHOD | DETAIL: FIELD \| CONSTR \| METHOD |  |


---


## trans.tpmap Class MummerMapper

```
java.lang.Object
  trans.tpmap.MummerMapper
```

---

``` public class MummerMapper extends java.lang.Object ```

Application to map 1lq files to fasta files.
Uses Mummer to map oligos to the fasta files.
Reverses the oligo sequences in the 1lq file prior to mapping with Mummer.

---

| **Field Summary** | |
| --- | --- |
| `static java.lang.String` | `controlChromosomeName`             String given to naming the control sequences. |


**Constructor Summary**

`MummerMapper(java.lang.String[] args)`


| **Method Summary** | |
| --- | --- |
| `boolean` | `appendMummerHits()`             Runs through mummer results and appends any hits to filtered1lq |
| `void` | `buildTPMap()`             Builds a tpmap file from a mummer results file |
| `java.util.ArrayList` | `fetchSameNumberLines(java.io.BufferedReader lines, int oligoLength)`             Loads the mummer results for one oligo into an ArrayList. |
| `static void` | `main(java.lang.String[] args)` |
| `void` | `makeFastaAndFilter()`             Makes a fasta file from a 1lq file, reverses the sequence of each oligo, drops control probes |
| `static void` | `printDocs()` |
| `void` | `processArgs(java.lang.String[] args)`             This method will process each argument and assign any new varibles |
| `boolean` | `runMummerExactMatcher(java.io.File genomeFastaFile)`             Runs MUMMER to find perfect matches |

**Methods inherited from class java.lang.Object**

`equals, getClass, hashCode, notify, notifyAll, toString, wait, wait, wait`

**Field Detail**

### controlChromosomeName

```
public static final java.lang.String controlChromosomeName
```

:   String given to naming the control sequences.

    **See Also:**: Constant Field Values


**Constructor Detail**

### MummerMapper

```
public MummerMapper(java.lang.String[] args)
```


**Method Detail**

### appendMummerHits

```
public boolean appendMummerHits()
```

:   Runs through mummer results and appends any hits to filtered1lq

---


### buildTPMap

```
public void buildTPMap()
```

:   Builds a tpmap file from a mummer results file

---


### fetchSameNumberLines

```
public java.util.ArrayList fetchSameNumberLines(java.io.BufferedReader lines,
                                                int oligoLength)
```

:   Loads the mummer results for one oligo into an ArrayList. Checks each mummer hit to ensure it is equal to the oligo length.
    No partial matches.

---


### runMummerExactMatcher

```
public boolean runMummerExactMatcher(java.io.File genomeFastaFile)
```

:   Runs MUMMER to find perfect matches

---


### makeFastaAndFilter

```
public void makeFastaAndFilter()
```

:   Makes a fasta file from a 1lq file, reverses the sequence of each oligo, drops control probes

---


### main

```
public static void main(java.lang.String[] args)
```

---


### processArgs

```
public void processArgs(java.lang.String[] args)
```

:   This method will process each argument and assign any new varibles

---


### printDocs

```
public static void printDocs()
```


---


|  |  |  |
| --- | --- | --- |
| **Overview** \| **Package** \| **Class** \| **Use** \| **Tree** \| **Deprecated** \| **Index** \| **Help** | |  |
| **PREV CLASS**   **NEXT CLASS** | **FRAMES**    **NO FRAMES**     **All Classes** |  |
| SUMMARY: NESTED \| FIELD \| CONSTR \| METHOD | DETAIL: FIELD \| CONSTR \| METHOD |  |


---
